# Opioids for chronic non-cancer pain: a protocol for a systematic review of randomized controlled trials

**DOI:** 10.1186/2046-4053-2-66

**Published:** 2013-08-21

**Authors:** Jason W Busse, Stefan Schandelmaier, Mostafa Kamaleldin, Sandy Hsu, John J Riva, Per Olav Vandvik, Ludwig Tsoi, Tommy Lam, Shanil Ebrahim, Bradley Johnston, Lori Oliveri, Luis Montoya, Regina Kunz, Anna Malandrino, Neera Bhatnagar, Sohail M Mulla, Luciane C Lopes, Charlene Soobiah, Anthony Wong, Norman Buckley, Daniel Sessler, Gordon H Guyatt

**Affiliations:** 1 Department of Anesthesia, McMaster University, Hamilton, Canada; 2 Department of Clinical Epidemiology and Biostatistics, McMaster University, Hamilton, Canada; 3 Academy of Swiss Insurance Medicine, University Hospital Basel, Basel, Switzerland; 4 Institute of Clinical Epidemiology and Biostatistics, University Hospital Basel, Basel, Switzerland; 5 Department of Family Medicine, McMaster University, Hamilton, Canada; 6 Norwegian Knowledge Centre for the Health Services, Oslo, Norway; 7 Accident and Emergency Department, Tseung Kwan O Hospital, Hong Kong, China; 8 Accident and Emergency Department, Tuen Mun Hospital, Hong Kong, China; 9 Department of Anesthesia and Pain Medicine, Hospital for Sick Children, Toronto, Canada; 10 Institute for Health Policy, Management and Evaluation, University of Toronto, Toronto, Canada; 11 Department of Dentistry, Santo Tomas University, Bogota, DC, Colombia; 12 Li Ka Shing Knowledge Institute, St. Michael’s Hospital, Toronto, Canada; 13 Health Sciences Library, McMaster University, Hamilton, Canada; 14 Pharmaceutical Sciences Post graduate Course, University of Sorocaba, UNISO, Sorocaba, Brazil; 15 Accident and Emergency Department, Pamela Youde Nethersole Eastern Hospital, Hong Kong, China; 16 Department of Outcomes Research, Cleveland Clinic, Cleveland, OH, USA

**Keywords:** Chronic pain, Opioid analgesics, Systematic review, Meta-analysis, Randomized controlled trials, Quality of life, Adverse effects

## Abstract

**Background:**

Opioids are prescribed frequently and increasingly for the management of chronic non-cancer pain (CNCP). Current systematic reviews have a number of limitations, leaving uncertainty with regard to the benefits and harms associated with opioid therapy for CNCP. We propose to conduct a systematic review and meta-analysis to summarize the evidence for using opioids in the treatment of CNCP and the risk of associated adverse events.

**Methods and design:**

Eligible trials will include those that randomly allocate patients with CNCP to treatment with any opioid or any non-opioid control group. We will use the guidelines published by the Initiative on Methods, Measurement, and Pain Assessment in Clinical Trials (IMMPACT) to inform the outcomes that we collect and present. We will use the Grading of Recommendations, Assessment, Development and Evaluation (GRADE) system to evaluate confidence in the evidence on an outcome-by-outcome basis. Teams of reviewers will independently and in duplicate assess trial eligibility, abstract data, and assess risk of bias among eligible trials. To ensure interpretability of our results, we will present risk differences and measures of relative effect for all outcomes reported and these will be based on anchor-based minimally important clinical differences, when available. We will conduct *a priori* defined subgroup analyses consistent with current best practices.

**Discussion:**

Our review will evaluate both the effectiveness and the adverse events associated with opioid use for CNCP, evaluate confidence in the evidence using the GRADE approach, and prioritize patient-important outcomes with a focus on functional gains guided by IMMPACT recommendations. Our results will facilitate evidence-based management of patients with CNCP and identify key areas for future research.

**Trial registration:**

Our protocol is registered on PROSPERO (CRD42012003023), http://www.crd.york.ac.uk/PROSPERO.

## Background

Chronic non-cancer pain (CNCP) includes any painful condition that persists for ≥3 months that is not associated with a diagnosis of cancer. The 2007/2008 Canadian Community Health Survey found that, among 57,660 respondents between the ages of 12 to 44 years, 10% reported CNCP. Prevalence increased with age and was significantly higher among those with lower education and among aboriginal populations [1]. More recently, in two reports Ramage-Morin and Ramage-Morin and Gilmour estimated that 38% of Canadian seniors in long-term care facilities and 27% of seniors living at home experience CNCP [2,3]. The National Center for Health Statistics estimates that 25% of the US population experiences CNCP [4] and Breivik *et al*. have reported a prevalence of 20% among the adult European population [5].

Chronic pain incurs a significant cost to society through lost work, decreased productivity, and high healthcare expenses. The cost of CNCP, taking into account direct medical expenses, costs of disability and lost productivity, is estimated at more than €200 billion per annum in Europe and US$150 billion per annum in the USA [6]. Chronic pain not caused by cancer is the primary cause of healthcare resource consumption and disability during adult working years [7].

The use of opioids for CNCP is considerable and continues to increase, particularly in North America [8-10], and Canada is currently the largest consumer of opioids in the world [11]. Canada, on a per capita basis, consumes five times the amount of prescription opioids used in the UK [12]. The Ontario Public Drug Benefit Plan reported more than a threefold increase in spending on oxycodone (OxyContin) over 5 years, from C$19.3 million in 2003/2004 to C$65 million in fiscal year 2008/2009. Province wide, the number of opioid prescriptions in Ontario rose from 3.7 to 4.7 million between 2005 and 2008 [13]. Similar trends have been reported in the USA: from 1980 to 2000, the prescription of opioids for CNCP increased from 2% to 9% of physician visits, which corresponds to 5.9 million visits in which opioids were prescribed for CNCP in 2000 [14]. Currently, opioids are the most commonly prescribed class of medication in the USA [15] and more than 3% of adults now receive long-term opioid therapy for CNCP [16].

Sullivan and colleagues have concluded that these trends have occurred without any significant change in the underlying population prevalence of CNCP and without new evidence for the efficacy of long-term opioid therapy [9]. These increases may be explained, in part, by aggressive marketing of sustained-release opioid formulations and public efforts to encourage clinicians to become more proactive in identifying and treating chronic pain [17-19].

Use of opioids for CNCP is not without risk. Studies have found a strong correlation between US states with high drug-poisoning mortality and those with high opioid consumption [20]. Opioid overdose is now the leading cause of unintentional death in the USA, having recently overtaken motor vehicle-related fatalities [21]. In 2010, opioids were responsible for at least 16,651 fatal drug overdoses in the USA; because 25% of all drug-related death certificates failed to record the type of drug responsible, this figure is a conservative estimate [22].

Patterns of prescribing opioids for CNCP vary widely between physicians. In 2006, Dhalla and colleagues examined drug-prescribing behaviors in Ontario and found that family physicians in the uppermost quintile (n = 1,978) had an average opioid-prescribing rate of 931.5 per 1,000 eligible patients during the study year, while physicians in the lowermost quintile (n = 1,977) had an average opioid prescription rate of 16.7 per 1,000 eligible patients. Therefore, family physicians in the uppermost quintile had an opioid-prescribing rate 56 times higher than physicians in the lowermost quintile [23]. This variation may be due to uncertainty regarding the relative benefits and harms of opioids in the management of CNCP, an issue that remains unclear despite the many published reviews and guidelines addressing this topic [24-31].

### Limitations of current evidence

The Canadian Guideline for Use of Opioids in Chronic Non-Cancer Pain [32] relied on a systematic review of opioids for CNCP [28] that has a number of limitations: (1) the review only included studies published up to May 2005; (2) the review only included trials published in English, Spanish or French, with the result that a number of eligible studies were not considered; (3) the authors report no measures of agreement for either their decisions on article eligibility or their determination of study quality; (4) the authors reported a single quality rating for each trial, which in turn is potentially misleading because risk of bias can differ between outcomes within trials; (5) the review used the Jadad scale to assess study quality [33], which has a number of limitations, including excessive consideration on reporting rather than performance, [34,35] and which has been superseded by other superior instruments, including the Cochrane risk of bias instrument [36]; (6) the authors collected only three outcome measures (pain, function, and adverse events), despite current recommendations that nine core outcome domains should be assessed in trials of CNCP in order to provide optimal information to patients, researchers, and clinicians [37-40]; (7) the authors reported results of their meta-analyses in standard deviation units using the standardized mean difference (SMD), an approach that is limited by vulnerability to differential variability in populations enrolled and challenges of interpreting the magnitude and importance of treatment effects [41,42]; and (8) the authors reported a subgroup analysis of strong versus less strong opioids that fails to meet important criteria for validity [43].

The review concluded, in part, that ‘for pain relief [non-opioid analgesics] were outperformed only by strong opioids’ [28], This positive result was based on two trials [44,45], one of which reported more responders for titrated morphine than for nortriptyline (52% vs 34%), but also a threefold greater loss to follow-up in the morphine group (19 of 71 vs 7 of 71) [44]. The second trial, an open-label study, showed small between-group differences in endpoint pain scores (scale 0 to 100) between naproxen alone (65.5), fixed-dose oxycodone (59.8), and titrated sustained-release morphine plus oxycodone (54.9) [45]. These limitations are not discussed in the review. The authors assessed the subgroup effect by testing the null hypothesis of no treatment effect in each of the relevant subgroups and claiming a subgroup effect when a significant effect was observed in the strong opioid subgroup, but not in the weak opioid subgroup. This strategy fails to address the real issue of subgroup analysis: can chance explain the apparent difference between subgroups? This question can be addressed with a formal test of interaction in which the null hypothesis assumes that the underlying effect across subgroups is the same. The authors did not report a test of interaction for their subgroup analysis. We propose to conduct an updated review of opioids for CNCP that will address these limitations.

## Methods and design

### Protocol and registration

Our protocol is registered on PROSPERO (CRD42012003023), http://www.crd.york.ac.uk/PROSPERO.

### Eligibility criteria

Eligible trials will include therapeutic trials that randomly allocate patients presenting with CNCP to an opioid analgesic or a non-opioid control. Chronic pain is defined as pain present for a duration of ≥3 months, or as defined by the study authors as chronic. We will also include chronic conditions characterized by remitting and relapsing symptoms, such as migraine-related headaches. If trials enroll a mix of cancer and CNCP patients, then to be eligible, they must report outcomes separately for the CNCP patients, or at least 90% of the patients must satisfy our criteria for CNCP. We will exclude trials that use opioids for diagnostic purposes or other non-therapeutic purposes (for example, to explore pain pathways). We will contact study authors if limitations in reporting lead to uncertainties in eligibility.

### Information sources

We will identify relevant randomized controlled trials (RCTs), in any language, by a systematic search of CINAHL, EMBASE, MEDLINE, AMED, HealthSTAR, PsycINFO, and the Cochrane Central Registry of Controlled Trials (CENTRAL), from inception of the databases. An experienced medical librarian (NB) has developed a sensitive search strategy for each individual database (see Appendix A for our MEDLINE search strategy). All eligible RCTs captured in previous reviews of opioids for CNCP [24-31] were captured in our search results, providing reassurance that our search strategy is robust. We will scan the bibliographies of all retrieved trials and other relevant publications, including reviews and meta-analyses, for additional relevant articles.

### Study selection

Using standardized forms, ten reviewers trained in health research methodology will work in pairs to screen, independently and in duplicate, titles and available abstracts of identified citations. We will acquire the full text publication of any article that is judged as potentially eligible by a paired review team. Six teams of reviewers will independently apply eligibility criteria to the full text of potentially eligible trials. Reviewers will resolve disagreement by consensus or, if a discrepancy remains, through discussion with an arbitrator (JWB or SMM). The ϕ (phi) statistic will provide a measure of interobserver agreement independent of chance regarding RCT eligibility.

### Data collection process and data items

We designed standardized forms and a detailed instruction manual that were used to create online data abstraction forms with DistillerSR (http://systematic-review.net/). Six teams of reviewers will extract data, independently and in duplicate, from each eligible study. Before starting data abstraction, reviewers will conduct calibration exercises to ensure consistency.

Data abstracted will include demographic information, methodology, intervention details, and outcome data on six core domains published by the Initiative on Methods, Measurement, and Pain Assessment in Clinical Trials (IMMPACT) [38-40] (Table 1). The members of IMMPACT recognize that their recommendations for outcomes in trials of CNCP were based on feedback from clinicians and researchers. In order to ensure that patients’ perspectives were considered, they conducted a series of focus groups (4 focus groups, including a total of 31 individuals) and an online survey of CNCP patients (68% response rate; 959 of 1,407) [37]. These exercises identified three additional core domains, which we will also collect for our review (Table 1). Data for all adverse outcomes will be collected as guided by Ioannidis and Lau [46]. We will resolve all disagreements by discussion to achieve consensus, and an arbitrator (JWB or SMM) will adjudicate unresolved disagreements.

**Table 1:** **Initiative on Methods, Measurement, and Pain Assessment in Clinical Trials (IMMPACT) core domains (1 to 6) **[38-40]**and additional patient reported core domains (7 to 9)**[37]

**Domain**	**Criteria**
1	Pain
2	Physical functioning (including quality of life)
3	Emotional functioning
4	Participant rating of improvement and satisfaction with treatment
5	Adverse symptoms and adverse events
6	Participant disposition (for example, adherence to the treatment regime and reasons for premature withdrawal from the trial)
7	Role functioning (that is, work and educational activities, social and recreational activities, home and family care)
8	Interpersonal functioning (that is, interpersonal relationships, sexual activities)
9	Sleep and fatigue

We will use the following rules for collection of outcome data: (1) we will abstract data from all patient-important outcomes (as guided by IMMPACT); (2) we will prioritize patient-important outcomes that are provided directly by patients; (3) If a patient-important outcome is reported by someone other than the patient (for example, clinician), or it unclear who reported a patient-important outcome, we will collect the data but make this distinction clear and perform a sensitivity analysis excluding non-patient and ‘unclear’ reporting from our analyses.

### Assessment of risk of bias in individual studies

Reviewers will assess risk of bias using a modified Cochrane risk of bias instrument. This instrument includes response options of ‘definitely or probably yes’ (assigned a low risk of bias) and ‘definitely or probably no’ (assigned a high risk of bias). We have previously shown this approach to be valid [47]. We will evaluate the following key domains: random sequence generation; allocation concealment; blinding of participants, healthcare professionals, data collectors, outcome assessors, and data analysts; and incomplete outcome data [48]. Reviewers will resolve disagreement by discussion and an arbitrator (JWB or SMM) will adjudicate any unresolved disagreements.

### Meta-analyses

To pool outcome data for trials that compare the same intervention with the same comparator, we will use random effects meta-analyses, which are conservative in that they consider both within and between study differences in calculating the error term used in the analysis [49,50].

We will use a number of approaches to improve the interpretability of results from our meta-analyses. For trials that report dichotomous outcomes, we will calculate the relative risk (RR) to inform relative effectiveness. We will also report the absolute risk reduction and acquire estimates of baseline risk from observational studies located through focused literature searches or, if not available, from the median of the control group from eligible RCTs. We will analyze categorical data as continuous if the distribution is relatively normal, or collapse data to a binary variable if not.

When pooling across trials that report continuous endpoints using the same instrument, we will calculate the weighted mean difference (WMD), which maintains the original unit of measurement and represents the average difference between groups [48]. The underlying principle of ‘weighting’ by inverse of variance is to accord more weight to studies that provide more information about the treatment effect. Once the WMD has been calculated, we will contextualize this value by noting the corresponding minimally important difference (MID): the smallest change in instrument score that patients perceive is important. We will conduct focused literature searches to identify anchor-based MIDs for relevant outcome measures.

#### Conversion of WMDs to interpretable units

We will prioritize anchor-based MIDs when available and calculate distribution based MIDs using the MID to SD ratio when they are not. For instance, if an MID is known for a commonly employed instrument, we will divide this MID by the SD of each of the trials that employed this measure. Based on this relationship, we will impute the MID for trials having employed instruments without an anchor-based MID on the basis of their SD and the ratio of MID to SD in the trials with established MIDs. We will refer to the ratio of the MID to the SD as the ‘SD ratio’ [51].

Within each pooled estimate, we will apply the median SD ratio from the trials with a known MID to the trials without a known MID. To do so, we will multiply the SD of each trial without a known MID by the median SD ratio value to arrive at the MID for that instrument. Having calculated an MID, we will divide the mean difference (MD) by the MID that was established for the instrument in order to obtain an estimate in MID units.

Contextualizing the WMD through the MID can be misleading because clinicians may interpret all mean effects below the MID as unimportant or presume important benefit for all patients when the mean difference exceeds the MID, which are not accurate conclusions. We will address this issue by assuming normal distributions of data and then calculating the proportions of participants in the intervention and control groups in each study that demonstrated an improvement greater than the MID [41]. The results are then pooled across studies.

If we only have post-test data (rather than magnitude of change), we will apply this approach if evidence exists regarding meaningful thresholds. For instance, if one knows that people with scores of less than 8 on the Hamilton rating scale for depression (HAM-D) are considered to be not depressed, one could examine the proportion of individuals below that threshold. If such meaningful thresholds do not exist, we will use post-test data and assume that the minimally important change within an individual corresponds, on average, to the MID between individuals. Making this assumption, one can calculate the difference in the proportion who benefit in intervention and control. To do this, we will take the mean value in the control group plus one MID unit, and calculate the proportion of patients in each group above that threshold.

#### Conversion of SMDs to interpretable units

For trials that use different continuous outcome measures that address the same underlying construct, a WMD cannot be calculated. We will therefore calculate the SMD. This step involves dividing the difference between the intervention and control means in each trial (that is, the mean difference) by the estimated between-person standard deviation (SD) for that trial. The SMD expresses the intervention effect in SD units, rather than the original units of measurement, with the value of a SMD depending on both the size of the effect (the difference between means) and the SD of the outcomes (the inherent variability among participants).

This common approach to pooling continuous outcome data can be problematic. If the heterogeneity of patients is different across studies, the SD will vary across studies. Therefore, given the same true difference in outcome between intervention and control groups, trials with more heterogeneous patients will show apparently, but spuriously, smaller effects than trials enrolling less heterogeneous patients. Furthermore, interpretation of the magnitude of effect when represented as SD units is not intuitive.

**Figure 1:**
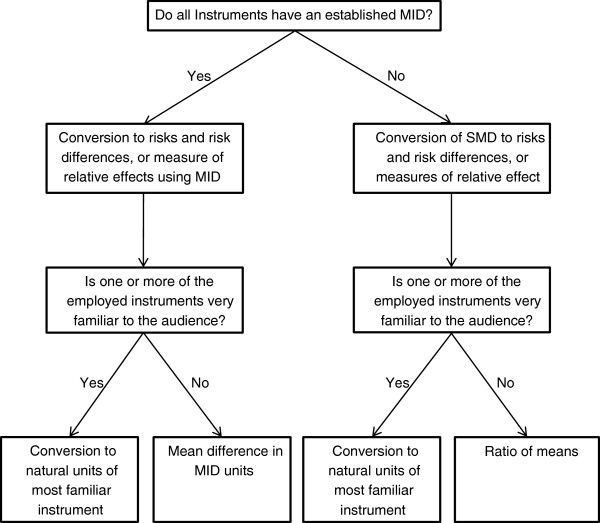
**Analysis strategy for pooling results of different continuous outcome measures **[38]**.**

In order to address these issues, we will contextualize the SMD value through MID units, which are not vulnerable to the distortions that varying heterogeneity of populations can create and are more interpretable to both clinicians and patients [41,52]. For outcome measures that have an established anchor-based MID, we will use this measure to convert the SMD into a RR. We will complement this presentation by either converting the SMD into natural units of a widely accepted instrument used to measure changes in the domain of interest (for example, visual analogue scale for pain) or, if such an instrument is not available, we will substitute the MID for the SD (denominator) in the SMD equation, which will result in more-readily interpretable MID units instead of SD units (Figure 1) [41]. Finally, we will provide a summary estimate of the proportion of patients who benefit from treatment across all studies.

#### Pooling crossover trials

We will pool crossover trials with parallel design using methods outlined in the Cochrane Handbook to derive effect estimates [48]. Specifically, we will perform a paired t test for each crossover trial if either of the following are available: (1) the individual participant data; (2) the mean and SD (or SE) of the participant-specific differences and between the intervention and control measurement; (3) the mean difference and one of the following: (i) a t statistic from a paired t test; (ii) a *P* value from a paired t test; (iii) a confidence interval from a paired analysis; or (4) a graph of measurements of the intervention arm and control arm from which we can extract individual data values (pending that the matched measurement for each individual can be identified) [48]. If these data are not available, we will approximate paired analyses by first calculating the MDs for the paired analyses (MD = M_E_ - M_C_) and the SE of the MD: SE (MD) = SD_diff_/√N, where N represents the number of participants in the trial, and SD_diff_ represents the SD of within-participant differences between the intervention and control measurements [48]. If the SE or SD of within-participant differences are not available, we will impute the SD using the methods outlined in the Cochrane Handbook [48].

### Assessment of heterogeneity and subgroup analyses

We will examine heterogeneity using both a χ^2^ test and the I^2^ statistic, the percentage of variability that is due to true differences between studies (heterogeneity) rather than sampling error (chance).

We have generated the following *a priori* hypotheses to explain variability between studies: (1) functional syndromes (for example, fibromyalgia) will show smaller effects versus objectively diagnosed conditions (for example, rheumatoid arthritis); (2) trials comparing opioids to placebo will show larger effects than trials using active comparators; (3) patients receiving disability benefits or involved in litigation will show smaller effects versus those that are not; (4) weaker opioids will show a smaller treatment effect than stronger opioids; (5) CNCP conditions characterized by remitting and relapsing symptoms will show smaller effects than conditions associated with constant pain; and (6) trials with higher risk of bias will show larger effects than trials with lower risk of bias. This last subgroup analysis will be completed on a risk of bias component-by-component basis, only if there is considerable variability within the risk of bias component. We will conduct tests of interaction [53] to establish if subgroups differ significantly from one another.

### Addressing missing participant data

We will use recently developed approaches to address missing participant data for dichotomous outcomes [54] and continuous outcomes [55]. When the primary analysis of our patient-important outcomes suggest important benefit, we will complete sensitivity meta-analyses to address missing participant data. For binary outcomes, if results are robust to a worst-case scenario (all intervention group participants with missing data suffered the outcome of interest, assuming the outcome is undesirable, all controls did not), we will conclude that missing data does not represent a risk of bias. If results are not robust to the typically implausible worst case, we will test progressively more extreme assumptions using methods proposed by Akl *et al*. [54]. For continuous outcomes, we will use a parallel approach proposed by Ebrahim *et al*. using four progressively more stringent imputation strategies that are based on observed outcomes among those followed-up in the individual trials included in the meta-analysis [55]. Important changes in results with such sensitivity analyses will be interpreted to represent serious risk of bias.

### Assessment of confidence in estimates

Reviewers will assess the confidence in effect estimates for all outcomes using the Grading of Recommendations, Assessment, Development and Evaluation (GRADE) rating system [56]. In the GRADE system of rating confidence in evidence for each outcome, randomized trials begin as high confidence evidence, but may be rated down by one or more of five categories of limitations: (1) risk of bias, (2) inconsistency, (3) indirectness, (4) imprecision, and (5) publication bias [57]. After considering these categories, the confidence in estimates for each outcome will be categorized as follows: ‘high’ confidence in evidence (we are very confident that the true effect lies close to that of the estimate of the effect); ‘moderate’ confidence in evidence (we are moderately confident in the effect estimate and the true effect is likely to be close to the estimate of the effect, but there is a possibility that it is substantially different); ‘low’ confidence in evidence (our confidence in the effect estimate is limited and the true effect may be substantially different from the estimate of the effect); and ‘very low’ confidence in evidence (we have very little confidence in the effect estimate and the true effect is likely to be substantially different from the estimate of effect) [58].

### Knowledge translation

The results of our proposed systematic review will be of interest to a broad audience, including patients diagnosed with CNCP, health professionals managing CNCP, employers, government healthcare benefits providers, insurers and compensation boards. We will involve relevant stakeholders from the onset of the review, in both research and dissemination activities, to improve the likelihood that the research results will be adopted and integrated into practice [59].

Members of our stakeholder committee will be invited to attend our planning meeting and share their input/advice with members of the review team. The stakeholder engagement will be led by a knowledge transfer associate, who will assist the research team to identify decision makers, conduct decision-maker meetings, and extract key messages from the review. Further, we are well connected with several European insurance groups in Switzerland, in the Netherlands, and with the European Union of Medicine in Assurance and Social Security (http://www.eumass.com), a European network of social insurance physicians. We plan to engage our colleagues in these groups to help promote dissemination to an international audience.

Our team also will engage in an end-of-study knowledge translation workshop. The purpose of this activity will be to share our findings with key relevant stakeholders (researchers, clinicians and decision makers), in order to: (1) identify future opportunities for dissemination, beyond traditional peer-reviewed publications, with our stakeholders; (2) discuss how to maximize uptake of our findings in patient education and clinical practice; and (3) determine future research directions. The overall goal of the workshop is to develop an agenda that will establish directions to develop and implement our research findings into practice.

The following strategies will be used to promote awareness of the stakeholder meeting findings according to the Ottawa Model of Research Use, in which information is tailored to specific audiences: (1) distribution of findings to all involved participants for further input, sharing within their organization, and sharing with their own stakeholders via newsletter, web site, or other methods; (2) presentation at relevant peer-reviewed and community conferences; and (3) publication in an open-source peer-reviewed journal. We anticipate that this meeting will identify new areas of inquiry for research and practice, such as the development of new educational tools for patients and clinicians. We also anticipate that new collaborations and networks will be created that will support the identified work going forward. Any groups identified through the meeting will be included as part of the report back to the stakeholders, in order to broadly disseminate the findings.

One member of our study team (NB) leads the Michael G DeGroote National Pain Centre that is located within McMaster University. This center has a mandate to update and distribute best practice guidelines in pain management, and specifically to update the Canadian Guideline for Use of Opioids in Chronic Non-Cancer Pain [31]. The results of our proposed systematic review will be used to update the Canadian Guideline, following which we will call a meeting of the National Faculty, a Canada wide group of 30 individuals representing organizations committed to the effective knowledge translation of the Canadian Guideline for Use of Opioids in Chronic Non-Cancer Pain. This meeting will establish working groups responsible for ensuring that the updated guideline is translated into practice, that appropriate teaching materials are created and disseminated, that legislative bodies are appropriately informed and that the impact of the guideline is evaluated.

## Discussion

Our review will evaluate both the effectiveness and the adverse events associated with opioid use for CNCP, evaluate confidence in the evidence using the GRADE approach [56], and prioritize patient-important outcomes with a focus on functional gains guided by IMMPACT recommendations [37-40]. With the assistance of our knowledge users, our results will guide an evidence-based use of opioids for patients with CNCP, identify key areas for future research and facilitate updating of the Canadian Guideline for Use of Opioids in Chronic Non-Cancer Pain [32].

## Appendix A: opioid and chronic non-cancer pain MEDLINE search strategy

Database: Ovid MEDLINE(R) in-process and other non-indexed citations and Ovid MEDLINE(R), 1950 to present.

### Search strategy

1. exp Analgesics, Opioid/

2. opioid$.mp.

3. (Asimadoline or Alvimopam or Fedotzine or Fentanyl).mp.

4. Hydrocodone.mp.

5. (Hydromorphone or Levorphanol or Meperidine or Morphine).mp.

6. (Oxycodone or Oxymorphone or Pentazocine or Propoxyphene).mp.

7. (Sufentanil or Tramadol).mp.

8. exp Codeine/

9. Codeine.mp.

10. or/1-9

11. exp Morphine/

12. morphine.tw.

13. morphia.mp.

14. ms contin.rn,mp.

15. oramorph sr.mp.

16. duramorph.mp.

17. 57-27-2.rn.

18. morphinene.mp.

19. anpec.mp.

20. duromorph.mp.

21. epimorph.mp.

22. miro.mp.

23. morfin.mp.

24. morfine.mp.

25. morphin.mp.

26. morphinium.mp.

27. morphium.mp.

28. opso.mp.

29. skenan.mp.

30. trama.mp.

31. (n-methylmorphine or n methylmorphine or isocodeine or ardinex).mp.

32. (phentanyl or fentanest or sublimaze or fentora).mp.

33. (duragesic or durogesic).mp.

34. (hydrocodon or dihydrocodeinone or dicodid or robidone or hydrocodeinonebitartrate or hydrocon).mp.

35. (dihydromorphinone or hydromorphon or palladone or laudacon or dilaudid).mp.

36. (codinovo or hycodan or hycon).mp.

37. (dihydromorphinone or hydromorphon or palladone or laudacon or dilaudid).mp.

38. (levodroman or levorphan or levo-dromoran or levodromoran).mp.

39. l dromoran.mp.

40. (pethidine or isonipecain or dolsin or dolosal or dolin or dolantin).mp.

41. (dolargan or lidol or lydol or Demerol or dolcontral).mp.

42. (dihydrohydroxycodeinone or oxycodeinon or dinarkon or eucodal).mp.

43. (hydroxycodeinon or oxiconum or oxycone or oxycontin).mp.

44. (pancodine or theocodin or dihydrone).mp.

45. (numorphan or talwin or lexir or fortral).mp.

46. (sulfentanyl or sulfentanil or sufenta).mp.

47. (tramadolhameln or tramadolor or tramadura or tramagetic or tramagit).mp.

48. (tramake or tramal or tramex or tramundin or trasedal).mp.

49. (ultram or zamudol or zumalgic or zydol or zytram).mp.

50. (adolonta or contramal or amadol or biodalgic or jutadol or nobligan or prontofort or takadol).mp.

51. (theradol or tiral or topalgic or tradol or tradolpuren or tradonal or tralgiol).mp.

52. (tramadorsch or biokanol or tramadin or tramadoc).mp.

53. exp narcotics/

54. or/11-53

55. 10 or 54

56. (chronic adj6 pain$).mp.

57. Chronic Disease/

58. exp Pain/

59. Low back pain.mp. or exp Back Pain/

60. backache$.mp.

61. Fibromyalgia.mp. or exp Fibromyalgia/

62. exp Whiplash Injuries/ or Whiplash.mp.

63. Irritable bowel syndrome.mp. or exp Irritable Bowel Syndrome/

64. Irritable colon.mp.

65. Temporomandibular joint syndrome.mp. or exp Temporomandibular Joint Dysfunction Syndrome/

66. Tension headache$.mp. or exp Tension-Type Headache/

67. Headache/

68. exp Cumulative Trauma Disorders/ or Repetitive strain syndrome.mp.

69. Osteoarthritis.mp. or exp Osteoarthritis/

70. Rheumatoid arthritis.mp. or exp Arthritis, Rheumatoid/

71. exp Diabetic Neuropathies/

72. diabetic neuropath$.mp.

73. Post herpetic neuralgia.mp. or exp Neuralgia, Postherpetic/

74. Postherpetic neuralgia.mp.

75. exp Phantom Limb/ or Phantom limb pain.mp.

76. exp Brachial Plexus Neuritis/ or cervicobrachial pain syndrome.mp.

77. globus syndrome.mp.

78. exp Headache Disorders/

79. neuropathic pain$.mp.

80. neuralgia.mp. or exp Neuralgia/

81. Pain Measurement/

82. diabetic neuropath$.mp.

83. polyneuropathies.mp. or exp Polyneuropathies/

84. polyneuropathy.mp.

85. or/56-84

86. randomized controlled trial$.mp.

87. randomized controlled trial.pt.

88. random allocation/

89. double-blind method/

90. single-blind method/

91. randomi?ed controlled trial$.mp.

92. controlled clinical trial.pt.

93. randomized controlled trial.pt.

94. ((singl$ or double$ or trebl$ or tripl$) adj25 (blind$ or mask$)).mp.

95. random$.mp.

96. placebo$.mp.

97. cross-over studies.sh.

98. latin square:.tw.

99. clinical trial.pt.

100. exp evaluation studies/

101. Retrospective Studies/ or follow up studies/ or prospective studies/

102. or/86-101

103. animals/ not humans/

104. 102 not 103

105. 55 and 85 and 104

106. 105 not ((acute or postoperative).ti,ab. not chronic.mp.)

107. 55 and 85 and comparative study/ and chronic.mp.

108. 107 not 103

109. 108 not ((acute or postoperative).ti,ab. not chronic.mp.)

110. 109 or 106

111. 110 not (exp neoplasms/ not chronic.mp.)

## Abbreviations

CNCP: Chronic non-cancer pain; GRADE: Grading of Recommendations Assessment, Development and Evaluation; IMMPACT: Initiative on Methods Measurement, and Pain Assessment in Clinical Trials; MID: Minimal important difference; SMD: Standardized mean difference.

## Competing interests

JWB acts as a consultant to Prisma Health Canada, a private incorporated company funded by employers and insurers that consults on and manages long-term disability claims. All other authors report no conflicts of interest, financial or otherwise.

## Authors’ contributions

JWB and GHG conceived the study design. NB designed the database-specific literature search strategies. AM and SH will retrieve all potentially eligible studies. JWB, SS, MK, SH, JJR, POV, LT, TL, SE, BJ, LO, LM, RK, SMM, LCL and AW will screen potentially eligible studies and abstract data from eligible trials. CS will oversee knowledge translation. LO, NB and DS provided oversight to ensure that our planned subgroup analyses and statistical pooling of effect sizes across patient populations, interventions, controls and outcome measures were clinically sensible. JWB and SS completed the first draft of the manuscript. All authors reviewed several drafts of the manuscript and approved the final version.
